# Confirmation Bias in Web-Based Search: A Randomized Online Study on the Effects of Expert Information and Social Tags on Information Search and Evaluation

**DOI:** 10.2196/jmir.3044

**Published:** 2014-03-26

**Authors:** Stefan Schweiger, Aileen Oeberst, Ulrike Cress

**Affiliations:** ^1^Knowledge Media Research CenterTuebingenGermany; ^2^University of TuebingenTuebingenGermany

**Keywords:** Web-based systems, prejudice, folksonomy, taxonomy, collaborative tagging, human information processing, psychotherapy, pharmacotherapy

## Abstract

**Background:**

The public typically believes psychotherapy to be more effective than pharmacotherapy for depression treatments. This is not consistent with current scientific evidence, which shows that both types of treatment are about equally effective.

**Objective:**

The study investigates whether this bias towards psychotherapy guides online information search and whether the bias can be reduced by explicitly providing expert information (in a blog entry) and by providing tag clouds that implicitly reveal experts’ evaluations.

**Methods:**

A total of 174 participants completed a fully automated Web-based study after we invited them via mailing lists. First, participants read two blog posts by experts that either challenged or supported the bias towards psychotherapy. Subsequently, participants searched for information about depression treatment in an online environment that provided more experts’ blog posts about the effectiveness of treatments based on alleged research findings. These blogs were organized in a tag cloud; both psychotherapy tags and pharmacotherapy tags were popular. We measured tag and blog post selection, efficacy ratings of the presented treatments, and participants’ treatment recommendation after information search.

**Results:**

Participants demonstrated a clear bias towards psychotherapy (mean 4.53, SD 1.99) compared to pharmacotherapy (mean 2.73, SD 2.41; *t*_173_=7.67, *P*<.001, *d*=0.81) when rating treatment efficacy prior to the experiment. Accordingly, participants exhibited biased information search and evaluation. This bias was significantly reduced, however, when participants were exposed to tag clouds with challenging popular tags. Participants facing popular tags challenging their bias (n=61) showed significantly less biased tag selection (*F*_2,168_=10.61, *P*<.001, partial eta squared=0.112), blog post selection (*F*_2,168_=6.55, *P*=.002, partial eta squared=0.072), and treatment efficacy ratings (*F*_2,168_=8.48, *P*<.001, partial eta squared=0.092), compared to bias-supporting tag clouds (n=56) and balanced tag clouds (n=57). Challenging (n=93) explicit expert information as presented in blog posts, compared to supporting expert information (n=81), decreased the bias in information search with regard to blog post selection (*F*_1,168_=4.32, *P*=.04, partial eta squared=0.025). No significant effects were found for treatment recommendation (*P*s>.33).

**Conclusions:**

We conclude that the psychotherapy bias is most effectively attenuated—and even eliminated—when popular tags implicitly point to blog posts that challenge the widespread view. Explicit expert information (in a blog entry) was less successful in reducing biased information search and evaluation. Since tag clouds have the potential to counter biased information processing, we recommend their insertion.

## Introduction

### Background

In the last decade, patients’ preferences have increasingly been taken into account when choosing a treatment for depression [1], which conforms to American Psychiatric Association guidelines [2]. Previous research has demonstrated, however, that laypeople hold beliefs about depression treatment that are partly inconsistent with scientific evidence. They believe, for instance, that psychotherapy is a more effective treatment for depression than pharmacotherapy [3,4]. In contrast to this, current scientific evidence demonstrates that pharmacotherapy and psychotherapy are nearly equally effective [5,6]. Consequently, the layperson’s beliefs are biased.

This paper investigates how biases like this one can be reduced. For our study, we chose the domain of depression treatment and made use of the psychotherapy bias. Specifically, we expected that laypeople’s bias towards psychotherapy leads to a confirmation bias in information search and evaluation. The confirmation bias refers to the robust findings that individuals tend to process information in a manner that confirms their pre-existing beliefs. Therefore, a confirmation bias in searching for information is not only of interest for depression treatment or the comparison of psychotherapy and pharmacotherapy, but for health-related information search in general. Individual convictions lead to one-sided information processing. When these convictions are not justified by scientific evidence, people run the risk of being misinformed.

Therefore, we investigated two factors that might reduce one-sided information processing. One of the most reliable and objective information sources on the Web is expert information. We tested whether facing explicit expert information would reduce the bias. Moreover, we were interested if aggregated expert information presented in tag clouds would reduce the bias as well.

### Blogs and Social Tagging

In the last decade, the Internet has become one of the most important sources for health-related information [7]. This phenomenon created the need to investigate the communication between experts and laypeople [8]. Blogs have been among the most effective applications for disseminating and discussing health-related topics by experts and a general audience. Blogs are authored by and targeted at laypeople as well as health professionals (eg, New York Times Well Blog [9], Harvard Health Blog [10]), and blogs often report current scientific studies, as well as the author’s personal opinion, which can be discussed by the public in the comments section. Moreover, blogs are among the crucial starting points for health-related online information search [11].

**Figure 1:**
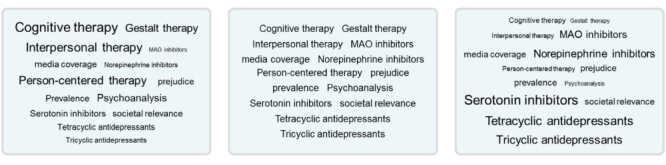
Tag cloud versions used in the study.

In order to provide an overview of the relevant content of a blog and to organize related blog posts, popular blogging sites such as Technorati, WordPress, or Counselling Resource [12-14] include tag clouds or tag lists [15]. We focus on tag clouds (Figure 1) because tag clouds provide implicit information on the popularity of topics. Tag clouds display different tags in varying font sizes, according to tag popularity. In broad folksonomies (eg, del.icio.us), which allow not only creators, but also recipients to tag digital artifacts, many people search for the same tags or provide the same tag for numerous blog posts. These co-occurring tags can be displayed in a tag cloud with varying font size, according to the number of co-occurrences.

Tags have two important functions. First, tags organize content. When people provide the same tag for different blog posts, blog posts with a common topic are quickly found via a common tag (eg, the topic with the tag “health” on WordPress [13]). Second, the font size of a tag reflects the popularity of the underlying concept. For example, Figure 1 demonstrates the three versions of a tag cloud with the same content, but different popularity of treatments for depressive disorders, used in the current study. Popular tags that represent treatments can be seen at a single glance [16,17].

Previous research on the perception of tag clouds has demonstrated that the popularity of tags (presented as tag size) influences information search and information evaluation [18,19]. Popular tags in a tag cloud, for instance, are more frequently selected and their resources more often consulted [20]. Popular tags not only guide navigation behavior but also information evaluation. Concepts represented by popular tags are rated as more typical of a domain [18]. Moreover, people align their cognitive concepts to the concepts represented by popular tags. After navigating with tags, people remember more popular concepts compared to less popular concepts [20,21].

### Confirmation Bias in Online Information Search

In order to investigate the confirmation bias in health-related information search, we chose the topic of depression treatment with pharmacotherapy or psychotherapy because previous research has demonstrated a discrepancy between laypeople’s beliefs and scientific evidence. As mentioned, psychotherapy is viewed to be more effective [3,4], whereas scientific evidence points to a comparable efficacy of both treatments [5,6]. We refer to this misconception as psychotherapy bias. Bias in our conception thus differs from personal preference in that it represents a systematic deviation from scientific knowledge and it describes subjective weightings of information. We expected that users’ information search is influenced by their belief that psychotherapy is more effective. Research from the confirmation bias has shown that people confirm their pre-existing beliefs by selecting information that supports those beliefs [22-26] (for an overview, see [27]).

The confirmation bias describes people’s need to confirm their beliefs and attitudes when engaged in search for online information [22,25]. Regarding the psychotherapy bias of laypeople, we expected that when people search for information, they would prefer information about the efficacy of psychotherapy over information about the efficacy of pharmacotherapy. This preference in turn strengthens their prior belief that psychotherapy is effective in treating depression.

Accordingly, our first hypothesis is that the psychotherapy bias—the conviction that psychotherapy is more effective than pharmacotherapy—leads to a confirmation bias in online information search where people prefer to select psychotherapy-related tags and content (H1).

If this confirmation bias determines information search, the question arises as to how the bias can be reduced. Research has shown that people perceive expert information as credible [27,28], and this leads people to align subsequent information search behavior. Therefore, we hypothesized that prior expert information that challenges pre-existing efficacy evaluations, compared to prior expert information that supports pre-existing evaluations, decreases biased information search (ie, tag selection and blog post selection; H2). Likewise, biased information search was expected to decrease with the provision of tag clouds that challenge pre-existent efficacy evaluations. That is, being exposed to tag clouds that have antidepressants as popular tags should decrease the predominant selection of psychotherapy-related tags and blog posts, in comparison to balanced tag clouds and tag clouds with psychotherapy as popular tags (H3). The same bias-reducing effects of challenging (vs supporting) prior expert information (H4) and challenging (vs balanced or supporting) tag clouds (H5) were expected with regard to the evaluation of information. Furthermore, we expected challenging (vs supporting) prior expert information (H6) and challenging (vs balanced or supporting) tag clouds (H7) to lead to a more frequent recommendation of pharmacotherapy.

## Methods

### Recruitment

Participants were recruited via two mailing lists, to which mostly university students from a broad range of disciplines had voluntarily enrolled. They were provided with a link that led them to a fully automated online survey. We reminded all participants twice via email to take part in the study. We did not use cookies or an IP (Internet protocol) check to detect or prevent multiple participation. However, all the provided email addresses were unique. There were no specific eligibility criteria with the exception of computer literacy as an implicit criterion. In order to have an 80% chance to detect a moderate effect (*f*=0.25), we would require 26 participants per group (a priori analysis of variance [ANOVA] power analysis conducted with G*Power 3.1.5; parameters set to *f*=0.25, 1-beta=.80, alpha=.05, numerator degrees of freedom=2, 6 groups; [29]). The study was conducted within a period of 10 weeks from December 2012 until March 2013 and was stopped after planned sample size was reached in all conditions.

**Figure 2:**
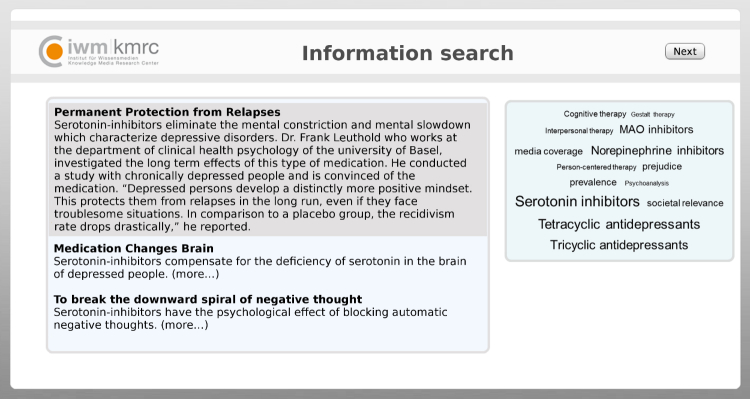
Screenshot of information search environment.

We outlined in the invitation mail that we were conducting a study on the treatment of depression, with the main task of rating short blog posts about different treatment options. We emphasized that participation would be voluntary, could be withdrawn at any point, and that the study would not cause harm of any kind. We also assured anonymity and the option to withdraw the data at the end of the study without providing reasons. Participants were informed about the duration of the study and the possibility to win €25 or €50 Amazon gift certificates. They were informed that by clicking the next button, they would provide informed consent. Moreover, they were asked to contact the experimenter (email was provided) in case of questions or considerations of any sort. There was no institutional affiliation presented in the invitation mail, but during the online study (see upper left part of Figure 2). Ethical approval was provided by the Ethical Committee of the Knowledge Media Research Center (LEK 2012/023).

### Design and Procedure

The study comprised a 2 (prior expert information: supporting, challenging) x 3 (tag popularity: psychotherapy, balanced, pharmacotherapy) between-subjects design. Participants were randomly assigned the following simple randomization procedures (computerized random numbers) to the different treatment groups, with the only restriction that a maximum of 35 individuals (who completed the study) were allowed per condition. We manipulated *prior expert information* by the content of the blogs that participants read before navigating in the tagging environment. Participants read either two blog posts highlighting the efficacy of psychotherapeutic treatment (supporting) of depressive disorders, or two blog posts highlighting the efficacy of pharmacotherapy (challenging).

As a second factor, we manipulated *tag popularity* by the font size of tags in the tagging environment. In the case of tag popularity, it is not a single resource that explicitly provides a statement regarding the efficacy of a treatment. Rather, the size of the tags implicitly provides insight into the popularity of treatments, as it is seen by experts. Either psychotherapy tags were displayed with a larger font size compared to pharmacotherapy tags, or pharmacotherapy tags were larger, or tags of both types of treatment had the same size (Figure 1, middle panel). Importantly, the tag-related blog posts presented during information search were the same across all conditions.

After the first two pages where participants were informed about the study and provided informed consent, the algorithm randomly assigned participants to one of the six conditions and a series of online forms followed. Participants filled out demographic data, followed by questionnaires (eg, prior beliefs about treatment efficacy, cf. measures section).

In the first phase of the experiment, participants read two blog entries. Participants were randomly assigned to read either two blog posts emphasizing the efficacy of psychotherapy (supporting the bias, n=93) or to read two blog posts emphasizing the efficacy of pharmacotherapy (challenging the bias, n=81) in the treatment of depressive disorders. The first blog entry reported that a large global network of “neurologists and psychologists” (expert information) agree on the efficacy of either pharmacotherapy or psychotherapy in the treatment of depression. The second blog entry presented the positive results of a neuroimaging evaluation study, arguing for the respective interpretation. Prior information was held constant, so the reasoning in both conditions was exactly the same; we interchanged only the terms antidepressants and psychotherapy. Note that no comparison to other types of treatment was provided in the blog posts. After each blog post, participants rated its persuasiveness.

After the first phase, participants were informed about the nature of tags and tag clouds. It was stated that tags describe and categorize online content, and an example of a tag cloud was shown. Participants were told that experts provided the tags in the following task. The more often a certain tag had been provided by these experts, the larger the tag in the cloud appeared. Therefore, participants were aware that large tags described popular topics among experts.

In the second phase of the experiment, participants searched for treatment-related information. The task for participants was to find useful information to provide information to a hypothetical friend who suffered from major depressive disorder. After the instructions, the information search environment appeared. Participants were randomly assigned to one of the three versions of a tag cloud (Figure 1). The tag cloud either supported psychotherapy bias (psychotherapeutic treatments popular, n=56), or it was neutral with respect to treatment popularity (all treatments equally popular, n=57), or it challenged psychotherapy bias (pharmacological treatments popular, n=61). Participants navigated with the static tag cloud to search information for psychotherapy and pharmacotherapy treatments. When participants clicked on a tag, three short related blog posts appeared to the left of the tag cloud. Blog posts were constant across all experimental conditions. Therefore, all participants had access to the same information. A pilot study (n*=*32) had assured that blog posts did not differ in persuasiveness, in order to rule out material effects. Tags in the cloud represented different types of treatment, and tag-related blog posts described the efficacy of the respective treatment. After 5 minutes, a stop button appeared at the upper right part of the screen. From this moment, participants could freely choose when to end the information search task. The timer was implemented in order to assure sufficient amount of navigational data.

At the end of the study, all participants were thoroughly debriefed and informed about the fact that the presented materials were not genuine materials and that tag clouds thus did not reflect actual scientific knowledge but had been experimentally designed.

### Materials

#### Content of Prior Expert Information

The two blog posts in the two different conditions of expert information contained matched main arguments for the efficacy of psychotherapy versus pharmacotherapy. Therefore, all blog posts in this study were fictitious. The first blog post in both conditions described the establishment of a database with scientific studies by an extensive and worldwide network of researchers. The second blog post in both conditions described the successful remediation of neuronal brain activity and brain structures, after treatment with either psychotherapy (supporting prior expert information) or pharmacotherapy (challenging prior expert information). Text length ranged from 98 to 118 words.

#### Tagging Environment

The tagging environment for information search consisted of two main sections (Figure 2). At the right side of the screen, 14 tags were presented. Five tags indicated psychotherapy, five tags indicated pharmacotherapy, and four tags were neutral with respect to treatment (media coverage, prejudice, prevalence, societal relevance; Figure 1). We varied tag popularity. In the *psychotherapy tags popular* condition, all psychotherapy tags were larger compared to pharmacotherapy tags. In the *pharmacotherapy tags popular* condition, all pharmacotherapy tags were larger compared to psychotherapy tags. In the *balanced tag popularity* condition, all tags had the same size.

At the left side of the screen in the tagging environment, for each tag, related blog posts were presented (Figure 2). Three blog posts were related to each tag. The content of the blog posts for pharmacotherapy (15 posts) and psychotherapy (15 posts) was held constant. We composed pairs of psychotherapy and pharmacotherapy blog posts, with the same main arguments and length (mean 76.8 words, SD 6.1) but different wording. Each post described a common symptom of depressive disorders (eg, psychomotor impairment) and scientific studies reported by an expert. The alleged experts concluded that the studies showed the efficacy of treatment by successfully reporting a remediation of the symptoms. All reported studies referred only to the efficacy of the respective treatment. There was no information available on the comparability of efficacy between pharmacotherapy and psychotherapy. A pilot study (n*=*32) assured that the blog posts had equal readability and that the persuasiveness and quality of all arguments did not differ within the pairs of blog posts about pharmacotherapy and psychotherapy. Initially, only the headline and the first sentence of each blog post were presented. In order to read the full blog post, participants had to click on the first sentence to expand the blog post.

The tagging environment displayed in the Web browser (programmed with Adobe Flash Builder) was developed by software developers at the Knowledge Media Research Center. The tagging environment was used for the first time; there were no changes of functionality during the period of data collection. Personal information (email address, demographic data) was stored separate from the survey data on a local server.

### Measures

#### Overview

Items of all the questionnaires were in fixed order; up to 7 items were displayed per screen. We implemented a completeness check so no items could be skipped by participants. Participants could not use a back button of the browser or within the survey. The measures are described in the order they appear in the experiment.

#### Prior Knowledge

Prior knowledge about depressive disorders was examined by 24 items regarding general knowledge (eg, false: “Women suffer from depressive disorders as often as men do”; true: “People suffering from diabetes are more likely to suffer also from depressive disorders compared to the general population”) and symptoms of depressive disorders according to the Diagnostic and Statistical Manual of Mental Disorders, 4th edition (DSM IV) and the International Classification of Diseases (ICD) 10 (eg, true: “Depressive disorders are often characterized by heightened or lowered appetite”; false: “People with a depressive disorder show an obsessive need for cleanliness and order”). The answer format had the three categories: true/false/I don’t know (Cronbach alpha=.72).

#### Evaluation

**Figure 3:**
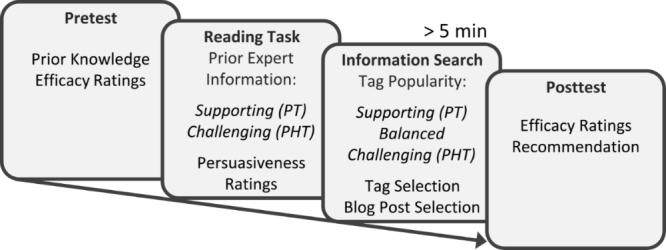
Study procedure.

Efficacy ratings were inquired for all the treatments that were presented prior to and after the experimental manipulations (see pre- and posttest, Figure 3). Five pharmacotherapy treatments and five psychotherapy treatments were rated on a 7-point scale ranging from 1 (not effective) to 7 (highly effective). Prior to the experimental manipulation, we also provided an additional category “I don’t know”, in case participants were not knowledgeable about the treatment in question (which was coded as 4 on the 7-point scale). A rating bias score was derived by subtracting the sum score of pharmacotherapy from psychotherapy efficacy ratings. If participants did not click on a tag, the respective treatment rating was excluded. The tagging environment produced log files that coded every click in the environment and the respective time. For the posttest ratings, we analyzed only treatments that were viewed by participants for at least 10 seconds according to the log files.

#### Persuasiveness Ratings of Blog Posts

After reading each of the two prior blog posts, participants rated the degree to which each blog post stated the efficacy of the presented treatment (either psychotherapy or pharmacotherapy) on a 7-point Likert scale (1=I agree not at all, 7=I completely agree). This rating served to ensure that the texts in both prior expert information conditions were equally convincing.

#### Information Search

In order to analyze the psychotherapy bias in information search, the number of selected pharmacotherapy tags was subtracted from the psychotherapy tags. Thus a positive value represented a searching bias towards psychotherapy. The same procedure was applied to the number of blog posts that participants read.

#### Recommendation

After the experimental manipulations, participants were asked to provide a treatment recommendation for a hypothetical friend. They were instructed to give reasons for the recommendation in about five sentences. Recommendations were coded from 1-5 (5: recommendation for psychotherapy only, 4: psychotherapy preferred, 3: combination therapy, 2: pharmacotherapy preferred, 1: pharmacotherapy only).

At the end of the study, participants had the opportunity to provide qualitative feedback through a feedback form.

### Statistical Methods

In order to test our main hypotheses, we conducted a 2 (prior expert information: supporting, challenging) x 3 (tag popularity: psychotherapy, balanced, pharmacotherapy) ANOVA with planned contrasts for the factor tag popularity. With additional *t* tests, we examined whether participants in the challenging *tag popularity* condition demonstrated any bias in information search at all.

## Results

### Participants and Dropout Analysis

**Figure 4:**
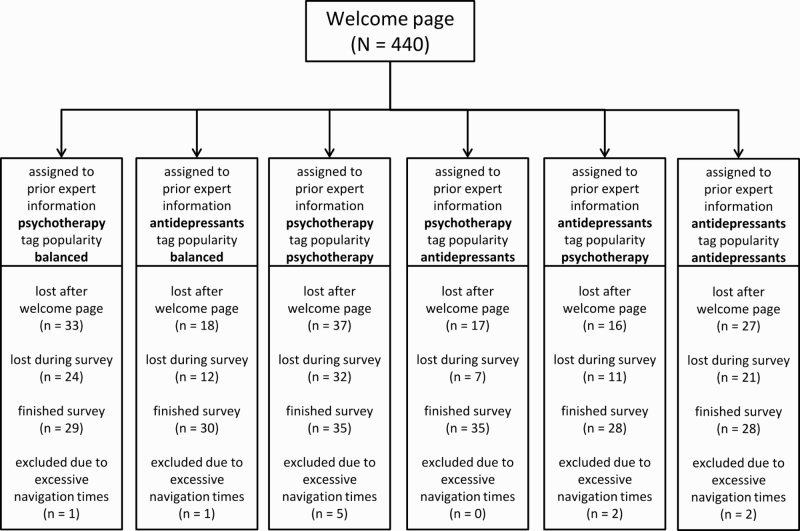
Participant flow diagram.

Initially, 440 individuals followed our invitation and started the online experiment. As can be seen in Figure 4, 33.6% (148/440) participants dropped out after the welcome page, and 24.3% (107/440) dropped out during the actual survey. The dropout during the survey is comparable to other online surveys [30]. In addition to these dropouts, we excluded a small number of participants 2.5% (11/440) due to excessive navigation times (see Figure 4). This was done in order to assure that the subsequent analysis of information search was not distorted by outliers. Excessive navigation times were detected using the conservative outlier labeling rule [31]. In order to make sure that our results were not specific for the complete cases, we analyzed tag selection and blog post selection for all participants who had participated up to this point and regardless of their navigation duration (50.9%, 224/440). The pattern of results was identical, which argues for the robustness of our findings. Our subsequent report will be based on those participants who completed the study and did not exhibit excessive navigation times (39.5%, 174/440).

Table 1 details the demographics and baseline characteristics of participants. Ages ranged from 16-62 years (mean 23.8, SD 3.8); 74.7% (130/174) were women. Regarding familiarity with the applications under investigation, 44.8% (78/174) stated that they were familiar with social tags, 26.4% (46/174) had knowingly assigned social tags on the Web, 67.2% (117/174) were reading blogs, and 13.8% (24/174) had authored a blog. Most of them were students (74.7%, 130/174) of a non–health care related subject (72.4%, 126/174). A minor proportion had health care related background knowledge due to their field of study (21.3%, 37/174): psychology, medicine, pharmacy, nursing care, molecular medicine, and neuroscience. It is noteworthy that we reran all analyses without participants from health care related subjects in order to test whether our results hold for laypeople, but the pattern of results was identical.

**Table 1:** Sample characteristics (N=174).

Characteristics		n	%
**Education**			
	Not yet graduated	130	74.7
	Graduated	43	24.7
	No higher education	1	0.6
**Field of study**			
	Health care related subject	37	21.3
	Non–health care related subject	126	72.4
	Not specified	11	6.3
**Age**			
	15-19	26	14.9
	20-24	97	55.7
	25-29	36	20.7
	30-39	10	5.7
	40-49	4	2.3
	62	1	0.6
Total		174	100

### Assuring Equivalence of Groups

First, we checked the equivalence of groups regarding participants’ *prior knowledge*. A 2 (prior expert information: supporting, challenging) x 3 (tag popularity: psychotherapy, balanced, pharmacotherapy) ANOVA showed no main effect of tag popularity (*F*_2,168_=2.32, *P*=.102, partial eta squared=0.027), and no significant effect of prior expert information (*F*_1,168_=3.63 *P*=.06, partial eta squared=0.021). Prior knowledge was not significantly related to any of the dependent variables (tag selection: *r*=–.04, *P*=.62; blog post selection: *r*=–.03, *P*=.66, efficacy rating: *r*=.03, *P*=.66, recommendation: *r*=.06, *P*=.47), nor was it a significant covariate, nor did prior knowledge as a covariate change the pattern of significance for each dependent variable in separate ANCOVAs. Therefore, we did not include prior knowledge as a covariate in the following analyses.

In order to assure equivalent *treatment intensity of prior expert information*, participants rated persuasiveness of both blog posts on a 7-point scale (1=I don’t agree, 7=I completely agree). There was no difference of the persuasiveness ratings between the prior pharmacotherapy expert information group (mean 5.86, SD 1.03) and the prior psychotherapy expert information group (mean 5.91, SD 1.11; *t*_173_=0.27, *P*=.79, *d*=0.08).

### Psychotherapy Bias

In the following analyses, we investigated whether participants showed a psychotherapy bias regarding pre-existent beliefs. To this end, we analyzed efficacy ratings of psychotherapy and pharmacotherapy that had been assessed prior to the information search. Efficacy ratings on a scale ranging from 1-7 showed that participants expressed strong superiority of psychotherapy (mean 4.53, SD 1.99) over pharmacotherapy (mean 2.73, SD 2.41; *t*_173_=7.67, *P*<.001, *d*=0.81) with regard to the treatment of depression. Thus the participants of our study clearly demonstrated a psychotherapy bias [3,4]. In the following sections, we will show how the bias influenced information processing and what factors affected the bias.

### Information Search

We first tested whether the psychotherapy bias emerges in information search (H1). This hypothesis was confirmed, since participants generally selected more psychotherapy tags (mean 4.66, SD 2.28) compared to pharmacotherapy tags (mean 3.87, SD 3.35; *t*_173_=2.83, *P*=.005, *d*=0.25). Further support was provided by the fact that participants selected more psychotherapy blog posts (mean 7.02, SD 4.47) compared to pharmacotherapy blog posts (mean 4.21, SD 3.97; *t*_173_=6.47, *P*<.001, *d*=0.66).

**Figure 5:**
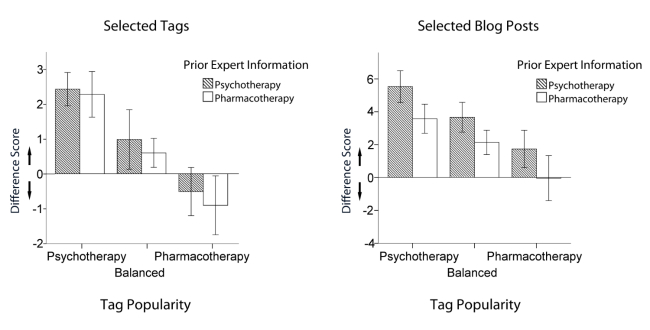
Information search bias (pharmacotherapy scores subtracted from psychotherapy scores; positive scores indicate a preference for psychotherapy over pharmacotherapy; negative scores indicate a preference of pharmacotherapy over psychotherapy).

Beyond demonstrating the biased information search behavior, we hypothesized that the psychotherapy bias is reduced by providing prior expert information (H2) and popular tags (H3) that challenge the psychotherapy bias. We will report two separate 2 (prior expert information: supporting, challenging) x 3 (tag popularity: psychotherapy, balanced, pharmacotherapy) ANOVAs for tag selection on one hand, and blog post selection on the other. With regard to *tag selection*, the analysis did not yield a significant main effect of *prior expert information* (*F*_1,168_=.32, *P*=.57, partial eta squared=0.002). There was no tendency of participants to prefer either pharmacotherapy or psychotherapy tags when prior expert information challenged or supported psychotherapy bias (Figure 5, left panel). There was, however, a significant main effect of *tag popularity* (*F*_2,168_=10.61, *P*<.001, partial eta squared=0.112). A polynomial contrast analysis showed that there was a linear trend of selection bias across the tag popularity conditions (*P*<.001; Figure 5, left panel). Psychotherapy tag selection was higher in the condition with psychotherapy tags being popular compared to the balanced condition (Cohen’s *d*=0.49) and the pharmacotherapy popular condition (Cohen’s *d*=0.85). The interaction between prior expert information and tag popularity (*F*_2,168_=.02, *P*=.98, partial eta squared<0.001) was not significant.

With regard to the second dependent measure of information search, *blog post selection*, a separate 2 x 3 ANOVA revealed a significant effect of *prior expert information* (*F*_1,168_=4.32, *P*=.04, partial eta squared=0.025). Reading a prior blog post that challenged the psychotherapy bias led participants to read more pharmacotherapy blog posts during their navigation in the tag cloud (Cohen’s *d*=0.30; Figure 5, right panel). The ANOVA also showed a main effect of *tag popularity* on biased *blog post selection* (*F*_2,168_=6.55, *P*=.002, partial eta squared=0.072). A polynomial contrast analysis showed that there was a linear trend of selection bias across the tag popularity conditions (*P*<.001; Figure 5, right panel). Psychotherapy blog post selection was higher in the psychotherapy tags popular condition compared to the balanced condition (Cohen’s *d*=0.38) and the pharmacotherapy popular condition (Cohen’s *d*=0.61). The interaction of prior expert information and tag popularity was not significant (*F*_2,168_=.02, *P*=.98, partial eta squared<0.001).

In an additional analysis, we exploratively examined whether participants in the challenging *tag popularity* condition exhibited any bias in information search at all. As indicated by *t* tests, this was not the case. Neither *tag selection* nor *blog post selection* were significantly biased: *P*s>14.

Taken together, we found evidence for a confirmation bias with participants selecting significantly more resources that were consistent with their previously held beliefs that psychotherapy is more effective. Our results also demonstrate, however, that this biased information selection can be significantly reduced. Whereas prior expert information reduced the biased selection of blog posts (but not of tags), tag popularity affected both measures of information search. Being exposed to a tag cloud that contained pharmacotherapy tags as the most popular ones did not only significantly decrease the biased search, but eventually eliminated the confirmation bias in that participants selected as many tags and resources of both treatment types. Hence, challenging tag clouds led to a balanced (ie, unbiased) information search.

### Evaluation of Information

With regard to information evaluation, we hypothesized that prior expert information (H4) that challenges the psychotherapy bias decreases biased evaluation of information, compared to prior expert information, which confirms psychotherapy bias. We also expected popular tags (H5) that challenge psychotherapy bias to reduce biased evaluation of information, compared to balanced tag popularity and even more compared to popular tags that support the bias. In order to analyze both hypotheses, we conducted a 2 (prior expert information: supporting, challenging) x 3 (tag popularity: psychotherapy, balanced, pharmacotherapy) ANOVA, with efficacy ratings as the dependent measure. The main effect of *prior expert information* (H4) on biased *efficacy rating* failed to reach conventional significance levels (*F*_1,168_=2.93, *P*=.09, partial eta squared=0.017). Prior expert information that challenged psychotherapy bias failed to significantly decrease biased information evaluation compared to prior expert information that confirms the bias.

**Figure 6:**
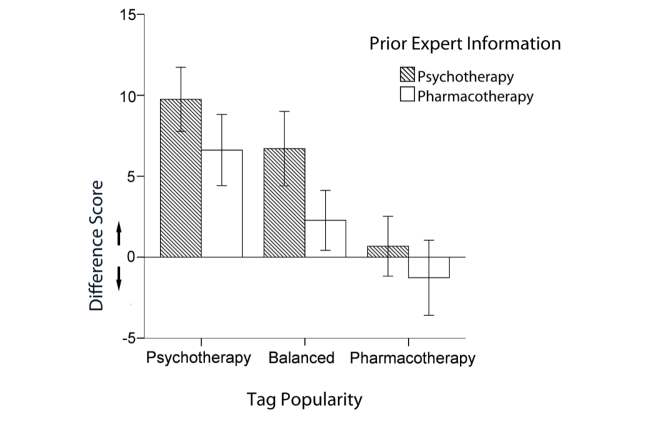
Efficacy ratings of blog posts (pharmacotherapy scores subtracted from psychotherapy scores; positive scores indicate a preference for psychotherapy over pharmacotherapy; negative scores indicate a preference of pharmacotherapy over psychotherapy).

Popularity of tags challenging psychotherapy bias, in contrast, decreased biased information evaluation as indicated by a significant main effect of *tag popularity* on *evaluation of information* (*F*_2,168_=8.48, *P*<.001, partial eta squared=0.092). A polynomial contrast analysis showed that there was a linear trend of evaluation bias across the tag popularity conditions (*P*<.001; Figure 6). Psychotherapy bias in treatment evaluation was higher in the psychotherapy tags popular condition compared to the balanced condition (Cohen’s *d*=0.35) and the pharmacotherapy popular condition (Cohen’s *d*=0.77). The interaction of prior expert information and tag popularity was not significant (*F*_2,168_=.18, *P*=.84, partial eta squared=0.002).

Further explorative analyses supported what can be derived from Figure 6 already. Efficacy ratings after the information search task were no longer biased in the challenging *tag popularity* condition (*t*_33_=0.37, *P*=.72 in the supporting *prior expert information* condition and *t*_25_=0.55, *P*=.59 in the challenging *prior expert information* condition).

In sum, our interventions were differentially successful in reducing the confirmation bias with regard to the evaluation of information. Whereas prior expert information failed to exert a significant influence, tag clouds with tags that challenged the psychotherapy bias not only reduced biased information evaluation, but eventually eliminated any bias. Efficacy ratings in this condition were thus eventually in line with scientific evidence.

### Recommendation

**Figure 7:**
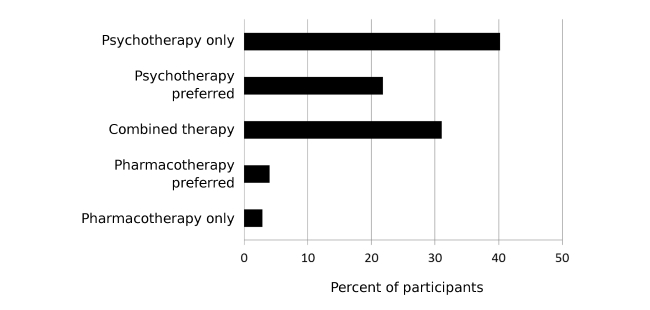
Treatment recommendation after experiment.

Beyond information selection and evaluation, we expected that *prior expert information* (H6), as well as *tag popularity* (H7) that challenges the psychotherapy bias, to decrease biased treatment recommendation for a hypothetical friend. We conducted an additional 2 (prior expert information: challenging, supporting) x 3 (tag popularity: psychotherapy, balanced, pharmacotherapy) ANOVA with treatment recommendation as the dependent variable. The results showed neither a significant main effect of tag popularity (*F*_2,168_=.22, *P*=.81, partial eta squared=0.003) nor a significant main effect of prior expert information (*F*_1,168_=.97, *P*=.33, partial eta squared=0.006). The interaction was also not significant (*F*_1,168_=.08, *P*=.92, partial eta squared=0.001). Overall, prior expert information and tag popularity had no effect on recommendation. Figure 7 shows that most of the participants recommended psychotherapy.

We conducted an exploratory qualitative analysis of the reasons for the treatment recommendation. Most of the participants did not provide any reasons but among those who did, the most frequently mentioned aspects regarded etiology or negative consequences of antidepressants. Specifically, 16.7% (29/174) participants argued for psychotherapy because they were convinced that biographical and social causes are crucial for the causation and treatment of depression. Another 10.3% (18/174) participants mentioned side effects, and 6.3% (11/174) reasoned that antidepressants are addictive. Finally, 4.6% (8/174) revealed that they believed that overcoming depression is an act of will or a personal responsibility.

## Discussion

### Principal Findings

This study investigated potential measures to decrease biased beliefs and their influence on information selection and information evaluation. To this end, we made use of laypeople’s (erroneous) convictions that psychotherapy is more effective in treating depression and examined whether this conviction guides online information search. In line with prior findings, participants did believe in the superiority of psychotherapeutic treatment and thus exhibited a psychotherapy bias. When searching for information online about the treatment of depressive disorders, participants showed a general bias towards selecting psychotherapy treatments compared to pharmacotherapy treatments.

We took two measures to reduce biased information processing. First, we exposed participants to expert information explicitly challenging the superiority of psychotherapy, by demonstrating the effectiveness of pharmacotherapy. This manipulation led participants to select fewer blog posts that were related to psychotherapy compared to the presentation of expert information supporting the effectiveness of psychotherapy. It did not affect, however, tag selection, and there was only a trend for it to exert an influence upon subsequent efficacy ratings. Hence, explicit expert information was only partially successful in reducing biased information processing.

Second, we attempted to decrease biased information processing by presenting participants with tag clouds in which the most popular tags referred to pharmacotherapy (vs psychotherapy). Consistent with our hypotheses, participants in the pharmacotherapy condition selected these popular pharmacotherapy tags more frequently and read more of the underlying blog posts. Moreover, treatment efficacy ratings were affected. In contrast to our expectations, however, we did not find any effects on treatment recommendations.

Although both manipulations had an impact upon search behavior and efficacy evaluation, the manipulations did not exert an impact on providing recommendations to other people. The gap between the efficacy ratings and treatment recommendations might be due to other beliefs people have with regard to both therapies, such as side effects [32,33]. Participants might be convinced that pharmacotherapy is effective, but they might still have feared detrimental side effects. The reasoning of participants supported this notion, as they frequently referred to side effects and even addictiveness of antidepressants when justifying their recommendation. This might indicate that even if a part of the beliefs changed (ie, the efficacy beliefs), other beliefs (eg, about side effects) still have a strong impact on the overall evaluation of a treatment. This is likely to be based on multiple aspects with efficacy being only one of them. Nevertheless, because our primary aim was to reduce laypeople’s misconceptions and to counter their biased information processing, we had primarily focused on treatment efficacy. After all, their beliefs had been shown to stand in contrast to scientific evidence. And it was due to this focus that all of our materials concerned treatment efficacy only. With regard to this misconception, however, our findings clearly argue for a success. Tag clouds with challenging popular tags were able to not only reduce biased information search and evaluation, but eventually led to an unbiased search and evaluation. That is, we were able to completely eliminate laypeople’s bias regarding treatment efficacy.

### Theoretical Implications

Previous research on confirmation bias has shown that people’s prior beliefs influence their information search in a way that they seek to confirm their beliefs [22,27]. The present study showed that implicit presentation of expertise is even more effective than the explicit one. Earlier research [21] showed that tag semantics and popularity determine individual information processing behavior. Likewise, previous studies successfully showed that social tags influence information selection, evaluation, incidental learning [18,19], and conceptual memory representations [20,21]. The findings of the current study extend existing evidence by showing that expert information exerts an even larger influence on users’ beliefs, if it is presented implicitly such as in tag clouds compared to explicit presentations as in blog posts alone. This finding has some practical implications.

### Practical Implications

In order to make people more aware of expert information and to overcome their individual biases, it seems to be useful to provide them with tag clouds. If these tag clouds challenge their subjective beliefs, users are motivated to select more popular tags (that are inconsistent with their own beliefs) and to read more information challenging their own views. This leads to a reduced confirmation bias, not just with regard to information search, but also with regard to evaluation.

A “correction” of subjective biases can only be achieved, however, if the information provided is not also biased. Thus, whether the effect that tag clouds have is really positive depends on the quality of tags and resources: does tag popularity really represent the scientific knowledge about a topic? In order to ensure that, it is important that people with high expertise provide the resources and tags. The provision of such expert information could be fostered if experts were encouraged to publish scientific studies in a style suitable for a broad audience, as this is already sufficient to reduce biased attitudes.

### Limitations

In the current study, we carefully balanced the quality of arguments for both types of treatment. We therefore provided information only about the efficacy of treatments, not about other aspects such as side effects, which would be specific for each treatment. For future studies, it may be desirable to test this in more depth by including diagnostic information with respect to relative efficacy of both treatment types (eg, information on treatments that are less effective compared to others or placebo), as well as providing information on side effects or other treatment-specific information.

Second, it must be pointed out that the present sample consisted mainly of university students or persons with a degree in higher education. Some of our participants had a health care related background. Our analyses showed, however, that the pattern of results was identical when these more knowledgeable participants were excluded. Hence, our findings should be valid with regard to laypeople. Nevertheless, future studies should also include participants without a higher education, as well as older persons.

### Conclusions

Our major aim in this study was to investigate whether people exhibit a biased online information search behavior that is guided by biased beliefs. We examined the biased perception of laypersons that psychotherapy is more effective than pharmacotherapy, when it comes to the treatment of depression [3,4]. We do not believe that our results are limited to the topic of depression or the pharmacological or psychological treatments. Rather, we would suggest that for any health-related issue involving different accounts or treatments, information challenging users’ prior knowledge and attitudes may increase their understanding of the topic in question [34,35].

## Multimedia Appendix 1

CONSORT-EHEALTH checklist V1.6.2 [36].

## Abbreviations

ANOVA: analysis of variance
DSM IV: Diagnostic and Statistical Manual of Mental Disorders, 4th edition
ICD-10: International Classification of Diseases
