# Supplementary material for: Confirmation Bias in Web-Based Search: A Randomized Online Study on the Effects of Expert Information and Social Tags on Information Search and Evaluation
Source: J Med Internet Res. 2014 Mar 26;16(3):e94. doi: 10.2196/jmir.3044 (PMC3978552; doi:10.2196/jmir.3044)
Supplement: Supplementary file 1 [file jmir_v16i3e94_app1.pdf]

# CONSORT-EHEALTH Checklist V1.6.2 Report

**(based on CONSORT-EHEALTH V1.6), available at [http://tinyurl.com/consort-ehealth-v1-6].**

3044

**Date completed**

3/7/2014 13:00:54

**by**

Stefan Schweiger

Confirmation Bias in Web-Based Search: A Randomized Online Study about the Effects of Expert Information and Social Tags on Information Search and Evaluation

## TITLE

**1a-i) Identify the mode of delivery in the title**

"Confirmation Bias in Web-Based Search: A Randomized Online Study about the Effects of Expert Information and Social Tags on Information Search and Evaluation"

**1a-ii) Non-web-based components or important co-interventions in title**

This item does not apply, as there were no non-web-based components or important co-interventions.

**1a-iii) Primary condition or target group in the title**

There is no primary condition in our case, since we investigate general aspects of information processing such as the confirmation bias. We therefore chose to mention the phenomenon of interest in the title—confirmation bias.

## ABSTRACT

**1b-i) Key features/functionalities/components of the intervention and comparator in the METHODS section of the ABSTRACT**

"First, participants read two alleged blog posts by experts that either challenged or supported the bias towards psychotherapy. Subsequently, participants searched for information about depression treatment in an online environment that provided more experts' blog posts about the effectiveness of treatments based on alleged research findings. These blogs were organized in a tag cloud; either psychotherapy tags were popular, or pharmacotherapy tags, or tags of both treatments were equally popular."

**1b-ii) Level of human involvement in the METHODS section of the ABSTRACT**

"174 participants completed a fully automated web-based study after we invited them via mailing lists."

**1b-iii) Open vs. closed, web-based (self-assessment) vs. face-to-face assessments in the METHODS section of the ABSTRACT**

"174 participants completed a fully automated web-based study after we invited them via mailing lists."

**1b-iv) RESULTS section in abstract must contain use data**

"Participants facing popular tags challenging their bias (N=61) showed significantly less biased tag selection ($F(2,168) = 10.61$, $P < .001$, $\eta p^2 = .112$), blog post selection ($F(2,168) = 6.55$, $P = .002$, $\eta p^2 = .072$) and treatment efficacy ratings ($F(2,168) = 8.48$, $P < .001$, $\eta p^2 = .092$), compared to bias-supporting tag clouds (N=56) and balanced tag clouds (N=57). Challenging (N=93) explicit expert information as presented in blog posts, compared to supporting expert information (N=81), decreased the bias in information search with regard to blog post selection ($F(1,168) = 4.32$, $P = .04$, $\eta p^2 = .025$). No significant effects were found for treatment recommendation ($Ps > .33$)."

**1b-v) CONCLUSIONS/DISCUSSION in abstract for negative trials**

": We conclude that the psychotherapy bias is most effectively attenuated—and even eliminated—when popular tags implicitly point to blog posts that challenge the widespread view. Explicit expert information (in a blog entry) was less successful in reducing biased information search and evaluation."

## INTRODUCTION

**2a-i) Problem and the type of system/solution**

"In the last decade, patients' preferences have been increasingly taken into account when choosing a treatment for depression [1] and guidelines by the American Psychiatric Association suggest to do so whenever possible [2]. Previous research has demonstrated, however, that laypeople hold beliefs about depression treatment that are partly inconsistent with scientific evidence. Laypersons believe, for instance, that psychotherapy is a more effective in treatment for depression than pharmacotherapy [3,4]. In contrast to this, current scientific evidence demonstrates that pharmacotherapy and psychotherapy are approximately equally effective [5,6]. Consequently, laypersons' beliefs are biased. They believe in psychotherapeutic superiority but this belief is not supported by scientific findings.

The present paper investigates how biases like this one can be reduced. For our study, we chose the domain of depression treatment and made use of the psychotherapy bias. Specifically, we expected that laypersons' bias towards psychotherapy leads to a confirmation bias in information search and evaluation. The confirmation bias refers to the robust finding that individuals tend to process information in a manner that confirms their pre-existing beliefs (see below). Therefore, a confirmation bias in searching for information is not only of interest for depression treatment or the comparison of psychotherapy and pharmacotherapy, but for health-related information search in general: Individual convictions lead to one-sided information processing. Particularly in the case that these convictions are not justified by scientific evidence, people run the risk of being misinformed.

Therefore, we investigated two factors that might reduce one-sided information processing. One of the most reliable and objective information sources on the web is expert information. We tested if facing explicit expert information would reduce the bias. Moreover, we were interested if aggregated expert information presented in tag clouds would reduce the bias as well."

**2a-ii) Scientific background, rationale: What is known about the (type of) system**

"In the last decade, the Internet has become one of the most important sources for health-related information [7], a phenomenon that created the need to investigate the communication between experts and laypeople [8]. Blogs have been among the most aspiring applications for disseminating and discussing health-related topics by experts and a general audience. Blogs are authored by and targeted at laypeople as well as health professionals (e. g., The New York Times Well Blog [9], Harvard Health Blog [10]), and blogs often report current scientific studies, as well as the author's personal opinion, which can be discussed by the public in the comments section. Moreover, blogs are among the crucial starting points for health-related online information search [11].
In order to provide an overview of the relevant content of a blog and to organize related blog posts, popular blogging sites such as Technorati, WordPress or Counselling Resource [12,13,14] include tag clouds or tag lists [15]. We focus on tag clouds (Figure 1), because tag clouds provide implicit information on the popularity of topics. Tag clouds are displaying different tags in varying font sizes, according to tag popularity. In broad folksonomies (e.g. del.icio.us), which allow not only creators, but also recipients to tag digital artifacts, many people search for the same tags, or provide the same tag for numerous blog posts. These co-occurring tags can be displayed in a tag cloud with varying font size, according to the number of co-occurrences.
Tags have two important functions. First, tags organize content. When people provide the same tag for different blog posts, blog posts with a common topic are quickly found via a common tag (e.g., the topic with the tag "health" on WordPress [13]). Second, the font size of a tag reflects the popularity of the underlying concept. For example, Figure 1 demonstrates the three versions of a tag cloud with the same content, but different popularity of treatments for depressive disorders, used in the current study. Popular tags that represent treatments can be seen at a single glance [16,17].
Previous research on the perception of tag clouds has demonstrated that the popularity of tags (presented as tag size) influences information search and information evaluation [18,19]. Popular tags in a tag cloud, for instance, are more frequently selected and their resources more often consulted [20]. Popular tags do not only guide navigation behavior but also information evaluation. Concepts represented by popular tags are rated as more typical of a domain [18]. Moreover, people align their cognitive concepts to the concepts represented by popular tags. After navigating with tags, people remember more popular concepts compared to less popular concepts [20,21]."

## METHODS

**3a) CONSORT: Description of trial design (such as parallel, factorial) including allocation ratio**

"Accordingly, our first hypothesis is that the psychotherapy bias—the conviction that psychotherapy is more effective than pharmacotherapy—leads to a confirmation bias in online information search in which people preferably select psychotherapy related tags and content (H1)."
"Therefore, we hypothesized that prior expert information that challenges pre-existing efficacy evaluations, compared to prior expert information that supports pre-existing evaluations, decreases biased information search (i.e., tag selection and blog post selection; H2). Likewise, biased information search was expected to decrease with the provision of tag clouds that challenge pre-existent efficacy evaluations. That is, being exposed to tag clouds that have antidepressants as popular tags should decrease the predominant selection of psychotherapy-related tags and blog posts, in comparison to balanced tag clouds and tag clouds with psychotherapy as popular tags (H3). The same bias-reducing effects of challenging (vs. supporting) prior expert information (H4) and challenging (vs. balanced or supporting) tag clouds (H5) were expected with regard to the evaluation of information. Furthermore, we expected challenging (vs. supporting) prior expert information (H6) and challenging (vs. balanced or supporting) tag clouds (H7) to lead to a more frequent recommendation of pharmacotherapy."

**3b) CONSORT: Important changes to methods after trial commencement (such as eligibility criteria), with reasons**

There were no such changes to report.

**3b-i) Bug fixes, Downtimes, Content Changes**

There were no such changes to report.

**4a) CONSORT: Eligibility criteria for participants**

"There were no specific eligibility criteria with the exception of computer literacy as an implicit criterion."

**4a-i) Computer / Internet literacy**

"There were no specific eligibility criteria with the exception of computer literacy as an implicit criterion."

**4a-ii) Open vs. closed, web-based vs. face-to-face assessments:**

"Participants were recruited via two mailing lists, in which mostly university students from a broad range of disciplines had voluntarily enrolled. They were provided with a link that led them to a fully automated online survey."

**4a-iii) Information giving during recruitment**

"We outlined in the invitation mail, that we were conducting a study on the treatment of depression, with the main task of rating short blog posts about different treatment options. We emphasized that participation would be voluntary, could be withdrawn at any point, and that the study would not cause harm of any kind. We also assured anonymity and the option to withdraw the data at the end of the study without providing reasons."

**4b) CONSORT: Settings and locations where the data were collected**

This item does not apply. Data were collected online, and we did not inquire the location of the participants.

**4b-i) Report if outcomes were (self-)assessed through online questionnaires**

As outlined in the measures section, all measures were self-assessed through the online questionnaires.

**4b-ii) Report how institutional affiliations are displayed**

"There was no institutional affiliation presented in the invitation mail, but during the online study (see Figure 3 upper left part of the screen)."

**5) CONSORT: Describe the interventions for each group with sufficient details to allow replication, including how and when they were actually administered**

**5-i) Mention names, credential, affiliations of the developers, sponsors, and owners**

"The tagging environment displayed in the web browser (programmed with Adobe® Flash® Builder®) was developed by software developers at the Knowledge Media Research Center."

**5-ii) Describe the history/development process**

"This software was developed and used for the first time,…"

**5-iii) Revisions and updating**

"...there were no changes of functionality during the period of data collection."

**5-iv) Quality assurance methods**

As outlined in the materials section, all information provided in the blog posts was fictitious (but believable as ensured by a pilot study), therefore the arguments were for psychotherapy and antidepressants were exactly balanced. Participants were informed of this fact within the context of a thorough debriefing at the end of the study.

**5-v) Ensure replicability by publishing the source code, and/or providing screenshots/screen-capture video, and/or providing flowcharts of the algorithms used**

Please find screenshots provided by Figures 1 and 3.

**5-vi) Digital preservation**

The study has been archived and all information (source code, screenshots, print version, demo pages) can be provided upon request. Also, the most important information can be seen in the screenshots in Figures 1 and 3. Moreover, the procedure of the entire experiment is reported in detail in the paper, making it easy for anyone to reproduce the study.

**5-vii) Access**

"Participants were recruited via two mailing lists, in which mostly university students from a broad range of disciplines had voluntarily enrolled. They were provided with a link that led them to a fully automated online survey."
"Participants were informed about the duration of the study and the possibility to win 25 or 50 Euro Amazon gift certificates."
**5-viii) Mode of delivery, features/functionalities/components of the intervention and comparator, and the theoretical framework**
"Content of Prior Expert Information
The two blog posts in the two different conditions of expert information contained matched main arguments for the efficacy of psychotherapy vs. pharmacotherapy. Therefore, all blog posts in this study were fictitious. The first blog post in both conditions described the establishment of a database with scientific studies by an extensive and worldwide network of researchers. The second blog post in both conditions described the successful remediation of neuronal brain activity and brain structures, after treatment with either psychotherapy (supporting prior expert information) or pharmacotherapy (challenging prior expert information). Text length ranged from 98 to 118 words.

Tagging Environment
The tagging environment for information search consisted of two main sections (Figure 3). At the right side of the screen 14 tags were presented. Five tags indicated psychotherapy, five tags indicated pharmacotherapy, and four tags were neutral with respect to treatment (media coverage, prejudice, prevalence, societal relevance; Figure 1). We varied tag popularity: In the psychotherapy tags popular condition, all psychotherapy tags were larger compared to pharmacotherapy tags. In the pharmacotherapy tags popular condition all pharmacotherapy tags were larger compared to psychotherapy tags. In the balanced tag popularity condition all tags had the same size.
At the left side of the screen in the tagging environment, for each tag, related blog posts were presented (Figure 3). Three blog posts were related to each tag. The content of the blog posts for pharmacotherapy (15 posts) and psychotherapy (15 posts) was held constant. We composed pairs of psychotherapy and pharmacotherapy blog posts, with the same main arguments and length ($M = 76.8$ words, $SD = 6.1$) but different wording. Each post described a common symptom of depressive disorders (e.g., psychomotor impairment), and scientific studies reported by an expert. The alleged experts concluded that the studies showed the efficacy of treatment by successfully reporting a remediation of the symptoms. All reported studies only referred to the efficacy of the respective treatment. There was no information available on the comparability of efficacy between pharmacotherapy and psychotherapy. A pilot study ($N=32$) assured that the blog posts had equal readability and that the persuasiveness and quality of all arguments did not differ within the pairs of blog posts about pharmacotherapy and psychotherapy. Initially, only the headline and the first sentence of each blog-post were presented. In order to read the full blog post, participants had to click on the first sentence to expand the blog post."
**5-ix) Describe use parameters**
In the current study, the use parameters would be the amount of time participants spent in information search.
"The task for participants was to find useful information to provide information to a hypothetical friend, who suffered from major depressive disorder."
"After 5 min, a stop button appeared at the upper right part of the screen. From this moment, participants could freely choose when to end the information search task."
**5-x) Clarify the level of human involvement**
"They [participants] were provided with a link that led them to a fully automated online survey."
**5-xi) Report any prompts/reminders used**
"We reminded all participants twice via email to take part in the study."
**5-xii) Describe any co-interventions (incl. training/support)**
Does not apply. In this study, no co-interventions were used.
**6a) CONSORT: Completely defined pre-specified primary and secondary outcome measures, including how and when they were assessed**
"Prior Knowledge
Prior knowledge about depressive disorders was inquired by 24 items regarding general knowledge (e.g., false: "Women suffer from depressive disorders as often as men do."; true: "People suffering from diabetes are more likely to suffer also from depressive disorders compared to the general population.") and symptoms of depressive disorders according to DSM IV and ICD 10 (e.g., true: "Depressive disorders are often characterized by heightened or lowered appetite"; false: "People with a depressive disorder show an obsessive need for cleanliness and order."). The answer format had the three categories true/false/I don't know (Cronbach's $\alpha = .72$).
Evaluation
Efficacy ratings were inquired for all the treatments that were presented prior to and after the experimental manipulations (cf. pre- and posttest, Figure 2). Five pharmacotherapy treatments and five psychotherapy treatments were rated on a 7-point scale ranging from 1 (not effective) to 7 (highly effective). Prior to the experimental manipulation we also provided an additional category "I don't know", in case participants were not knowledgeable about the treatment in question (which was coded as 4 on the 7-point scale). A rating bias score was derived by subtracting the sum score of pharmacotherapy from psychotherapy efficacy ratings. If participants did not click on a tag, the respective treatment rating was excluded. The tagging environment produced log files that coded every click in the environment and the respective time. For the posttest ratings we only analyzed treatments that were viewed by participants for at least 10 seconds according to the log files.
Persuasiveness Ratings of Blog Posts
After reading each of the two prior blog posts, participants rated the degree to which each blog post stated the efficacy of the presented treatment (either psychotherapy or pharmacotherapy) on a 7-point Likert scale (1 = I agree not at all, 7 = I completely agree). This rating served to ensure that the texts in both prior expert information conditions were equally convincing.
Information Search
In order to analyze the psychotherapy bias in information search, the number of selected pharmacotherapy tags was subtracted from the psychotherapy tags. Thus a positive value represented a searching bias towards psychotherapy. The same procedure was applied to the number of blog posts that participants read.
Recommendation
After the experimental manipulations, participants were asked to provide a treatment recommendation for a hypothetical friend. They were instructed to give reasons for the recommendation in about five sentences. Recommendations were coded from 1 to 5 (5: recommendation for psychotherapy only, 4: psychotherapy preferred, 3: combination therapy, 2: pharmacotherapy preferred, 1: pharmacotherapy only)."
**6a-i) Online questionnaires: describe if they were validated for online use and apply CHERRIES items to describe how the questionnaires were designed/deployed**
The questionnaires of the present study were not previously validated for online use. However, we provided Cronbach's Alpha measures to check reliability. We included informatation on all the CHERRIES items.
**6a-ii) Describe whether and how "use" (including intensity of use/dosage) was defined/measured/monitored**

Intensity of dosage/use is in the current study related to the subjective persuasiveness of prior expert information on the one hand, and the extent to which the tag popularity had impact on navigation behavior on the other hand.
"Persuasiveness ratings of blog posts
After reading each of the two prior blog posts, participants rated the degree to which each blog post stated the efficacy of the presented treatment (either psychotherapy or pharmacotherapy) on a 7-point Likert scale (1 = I agree not at all, 7 = I completely agree). This rating served to ensure that the texts in both prior expert information conditions were equally convincing."
"In order to analyze the psychotherapy bias in information search, the number of selected pharmacotherapy tags was subtracted from the psychotherapy tags. Thus a positive value represented a searching bias towards psychotherapy. The same procedure was applied to the number of blog posts that participants read."

**6a-iii) Describe whether, how, and when qualitative feedback from participants was obtained**

"At the end of the study, participants had the opportunity to provide qualitative feedback through a feedback form."

**6b) CONSORT: Any changes to trial outcomes after the trial commenced, with reasons**

There were no changes to trial outcomes after the survey went online.

**7a) CONSORT: How sample size was determined**

**7a-i) Describe whether and how expected attrition was taken into account when calculating the sample size**

"In order to have an 80% chance to detect a moderate effect ($f = 0.25$), we would require 26 participants per group (a priori ANOVA power analysis conducted with G*Power 3.1.5; parameters set to $f = .25$, $1–\beta = .80$, $\alpha = .05$, numerator df = 2, 6 groups; [35])."

**7b) CONSORT: When applicable, explanation of any interim analyses and stopping guidelines**

This item does not apply. There were no interim analyses conducted.

**8a) CONSORT: Method used to generate the random allocation sequence**

"Participants were randomly assigned following simple randomization procedures (computerized random numbers) to the different treatment groups,..."

**8b) CONSORT: Type of randomisation; details of any restriction (such as blocking and block size)**

The study comprised of a 2 (prior expert information: supporting, challenging) x 3 (tag popularity: psychotherapy, balanced, pharmacotherapy) between subjects design. Participants were randomly assigned following simple randomization procedures (computerized random numbers) to the different treatment groups, with the only restriction that a maximum of 35 individuals (who completed the study) were allowed per condition."

**9) CONSORT: Mechanism used to implement the random allocation sequence (such as sequentially numbered containers), describing any steps taken to conceal the sequence until interventions were assigned**

"Participants were randomly assigned following simple randomization procedures (computerized random numbers) to the different treatment groups,..."

**10) CONSORT: Who generated the random allocation sequence, who enrolled participants, and who assigned participants to interventions**

We reported that our software developers programmed the fully automated random allocation sequence. Participants enrolled themselves in mailing lists, after they were asked to do by our research assistants. And the authors of the present study sent invitation mails to the participants.
"Participants were recruited via two mailing lists, in which mostly university students from a broad range of disciplines had voluntarily enrolled. They were provided with a link that led them to a fully automated online survey."
"After the first two pages where participants were informed about the study and provided informed consent, the algorithm randomly assigned participants to one of the six conditions and a series of online forms followed."

**11a) CONSORT: Blinding - If done, who was blinded after assignment to interventions (for example, participants, care providers, those assessing outcomes) and how**

**11a-i) Specify who was blinded, and who wasn't**

As the current online study implies: Experimenters knew about all experimental conditions. They were not involved in the assignment procedure, however, and did not interact with participants.
Participants did not know about other experimental conditions prior to debriefing at the very end of the study.

**11a-ii) Discuss e.g., whether participants knew which intervention was the "intervention of interest" and which one was the "comparator"**

As mentioned above, participants did not know about other experimental conditions prior to debriefing at the very end of the study. Also, since this study was fully automated they did not interact with experimenters.

**11b) CONSORT: If relevant, description of the similarity of interventions**

This item does not apply to the present online study.

**12a) CONSORT: Statistical methods used to compare groups for primary and secondary outcomes**

"In order to test our main hypotheses we conducted A 2 (prior expert information: supporting, challenging) x 3 (tag popularity: psychotherapy, balanced, pharmacotherapy) ANOVA with planned contrasts for the factor tag popularity."

**12a-i) Imputation techniques to deal with attrition / missing values**

"In order to make sure that our results were not specific for the complete cases we analyzed tag selection and blog post selection for all participants who had participated up to this point and irrespective of their navigation duration (N=224). The pattern of results was identical, which argues for the robustness of our findings. For the sake of clarity, however, our subsequent report will be based on those participants, who completed the study and did not exhibit excessive navigation times."

**12b) CONSORT: Methods for additional analyses, such as subgroup analyses and adjusted analyses**

"With additional t-tests, we examined whether participants in the challenging tag popularity condition demonstrated any bias in information search at all."

## RESULTS

**13a) CONSORT: For each group, the numbers of participants who were randomly assigned, received intended treatment, and were analysed for the primary outcome**

This information was provided in the participant flow diagram (Figure 4).

**13b) CONSORT: For each group, losses and exclusions after randomisation, together with reasons**

This information was provided in the participant flow diagram (Figure 4).

**13b-i) Attrition diagram**

We did not provide an attrition diagram, as we think it would not provide very helpful information for the current study.

**14a) CONSORT: Dates defining the periods of recruitment and follow-up**

"The study was conducted within a period of 10 weeks from December 2012 until March 2013..."

**14a-i) Indicate if critical "secular events" fell into the study period**

To the best of our knowledge, there were no pertinent secular events.

**14b) CONSORT: Why the trial ended or was stopped (early)**

"In order to have an 80% chance to detect a moderate effect ($f = 0.25$), we would require 26 participants per group (a priori ANOVA power analysis conducted with G*Power 3.1.5; parameters set to $f = .25$, $1–\beta = .80$, $\alpha = .05$, numerator df = 2, 6 groups; [35]). The study was conducted within a period of 10 weeks from December 2012 until March 2013, and was stopped after planned sample size was reached in all conditions."

**15) CONSORT: A table showing baseline demographic and clinical characteristics for each group**

Information on participant characteristics is presented in Table 1.

**15-i) Report demographics associated with digital divide issues**
"Table 1 details the demographics and baseline characteristics of participants. Age ranged from 16 to 62 years (M = 23.8, SD = 3.8); 130 (74.7%) were women. Regarding familiarity with the applications under investigation, 78 (44.8%) stated that they were familiar with social tags, 46 (26.4%) knowingly assigned social tags on the web, 117 (67.2%) read blogs, and 24 (13.8%) authored a blog. Most of them were students (74.7%) of a non-health care related subject (72.4%). A minor proportion had health care related background knowledge due to their field of study (21.3%)."
**16a) CONSORT: For each group, number of participants (denominator) included in each analysis and whether the analysis was by original assigned groups**
**16-i) Report multiple "denominators" and provide definitions**
This item does not apply, as there were no multiple denominators in the current study.
**16-ii) Primary analysis should be intent-to-treat**
This item does not apply, as there were no multiple denominators in the current study.
**17a) CONSORT: For each primary and secondary outcome, results for each group, and the estimated effect size and its precision (such as 95% confidence interval)**
The paper reports Cohen's d as well as partial eta squared effect sizes. Precision measures are given by standard deviations along with means and standard errors in diagrams.
**17a-i) Presentation of process outcomes such as metrics of use and intensity of use**
Please find figure 5 as well as the navigation analyses.
"We first tested whether the psychotherapy bias emerges in information search (H1). This hypothesis was confirmed, since participants generally selected more psychotherapy tags (M = 4.66, SD = 2.28) compared to pharmacotherapy tags (M = 3.87, SD = 3.35; t(173) = 2.83, P = .005, d = .25). Further support was provided by the fact that participants selected more psychotherapy blog posts (M = 7.02, SD = 4.47) compared to pharmacotherapy blog posts (M = 4.21, SD = 3.97; t(173) = 6.47, P < .001, d = .66)."
**17b) CONSORT: For binary outcomes, presentation of both absolute and relative effect sizes is recommended**
We did not conduct inferential statistics based on binary dependent measures.
**18) CONSORT: Results of any other analyses performed, including subgroup analyses and adjusted analyses, distinguishing pre-specified from exploratory**
We mentioned that we also did a reanalysis without participants who had a healthrelated background.
"In an additional analysis we exploratively examined whether participants in the challenging tag popularity condition exhibited any bias in information search at all. As indicated by t-tests, this was not the case. Neither tag selection, nor blog post selection were significantly biased, Ps > 14."
"Further explorative analyses supported what can be derived from Figure 6 already: Efficacy ratings after the information search task were no longer biased in the challenging tag popularity condition (t(33) = 0.37, P = .72 in the supporting prior expert information condition and t(25) = 0.55, P = .59 in the challenging prior expert information condition)."
**18-i) Subgroup analysis of comparing only users**
Subgroup analyses played a minor role in the current paper, therfore we did not discuss it in detail.
**19) CONSORT: All important harms or unintended effects in each group**
To the best of our knowledge, there were no harms in this online study. We thoroughly debriefed participants about false information provided in the study.
**19-i) Include privacy breaches, technical problems**
There were neither technical problems nor privacy breaches.
**19-ii) Include qualitative feedback from participants or observations from staff/researchers**
We did not include qualitative feedback as it did not provide deeper insight in the results of the current study.

## DISCUSSION

**20) CONSORT: Trial limitations, addressing sources of potential bias, imprecision, multiplicity of analyses**
**20-i) Typical limitations in ehealth trials**
"In the current study we carefully balanced the quality of arguments for both types of treatment. We therefore only provided information about the efficacy of treatments, not about other aspects such as side effects, which would be specific for each treatment. For future studies it may be desirable to test this in more depth by including diagnostic information with respect to relative efficacy of both treatment types (e.g. information on treatments that are less effective compared to others or placebo), as well as providing information on side effects or other treatment-specific information."
**21) CONSORT: Generalisability (external validity, applicability) of the trial findings**
**21-i) Generalizability to other populations**
"Second, it must be pointed out that the present sample consisted mainly of university students or persons with a degree in higher education. Some of our participants had a health-care related background. Our analyses showed, however, that the pattern of results was identical when these more knowledgeable participants were excluded. Hence, our findings should be valid with regard to laypeople. Nevertheless, it would be desirable for future studies to also include participants without a higher education, as well as older persons."
**21-ii) Discuss if there were elements in the RCT that would be different in a routine application setting**
This item is not applicable to the current study.
**22) CONSORT: Interpretation consistent with results, balancing benefits and harms, and considering other relevant evidence**
**22-i) Restate study questions and summarize the answers suggested by the data, starting with primary outcomes and process outcomes (use)**
"This study investigated potential measures to decrease biased beliefs and their influence on information selection and information evaluation. To this end we made use of laypersons' (erroneous) convictions that psychotherapy is more effective in treating depression and examined whether this conviction guides online information search. In line with prior findings, participants did believe in the superiority of psychotherapeutic treatment and thus exhibited a psychotherapy bias. When searching for information online about the treatment of depressive disorders, participants showed a general bias towards selecting psychotherapy treatments compared to pharmacotherapy treatments.
We took two measures to reduce biased information processing. First, we exposed participants to expert information explicitly challenging the superiority of psychotherapy, by demonstrating the effectiveness of pharmacotherapy. This manipulation led participants to select fewer blog posts that were related to psychotherapy compared to the presentation of expert information supporting the effectiveness of psychotherapy. It did not affect, however, tag selection and there was only a trend for it to exert an influence upon subsequent efficacy ratings. Hence, explicit expert information was only partially successful in reducing biased information processing.
Second, we attempted to decrease biased information processing by presenting participants with tag clouds in which the most popular tags referred to pharmacotherapy (vs. psychotherapy). Consistent with our hypotheses, participants in the pharmacotherapy condition selected these popular pharmacotherapy tags more frequently and read more of the underlying blog posts. Moreover, treatment efficacy ratings were affected. In contrast to our expectations, however, we did not find any effects on treatment recommendations. "
**22-ii) Highlight unanswered new questions, suggest future research**

"For future studies it may be desirable to test this in more depth by including diagnostic information with respect to relative efficacy of both treatment types (e.g. information on treatments that are less effective compared to others or placebo), as well as providing information on side effects or other treatmentspecific information."
"Nevertheless, it would be desirable for future studies to also include participants without a higher education, as well as older persons."
"We do not believe that our results are limited to the topic of depression or the pharmacological or psychological treatments. Rather, we would suggest that for any health-related issue involving different accounts or treatments, it should be the case that information challenging users' prior knowledge and attitudes may increase their elaboration with the topic in question [33,34]."

## Other information

**23) CONSORT: Registration number and name of trial registry**

Not applicable. The present study was not registered as RCT; it was a randomized online study on information search behavior.

**24) CONSORT: Where the full trial protocol can be accessed, if available**

Not applicable.

**25) CONSORT: Sources of funding and other support (such as supply of drugs), role of funders**

"The current study was funded by the internal budget of the Knowledge Media Research Center."

**X26-i) Comment on ethics committee approval**

"Ethical approval was provided by the Ethical Committee of the Knowledge Media Research Center (LEK 2012/023)."

**x26-ii) Outline informed consent procedures**

"They were informed that by clicking the next button, they would provide informed consent."

**X26-iii) Safety and security procedures**

"Moreover, they were asked to contact the experimenter (email was provided) in case of questions or considerations of any sort."

**X27-i) State the relation of the study team towards the system being evaluated**

This was neither a study that evaluated software nor was it externally funded.
